# Paget’s Disease Mimicking Bone Metastasis in a Patient with Neuroendocrine Tumor on ^68^Ga-DOTANOC PET/CT

**DOI:** 10.5334/jbr-btr.903

**Published:** 2016-07-11

**Authors:** Naciye Sinem Gezer, Ali Balcı, Özhan Özdoğan, Dinç Özaksoy

**Affiliations:** 1 Dokuz Eylul University Faculty of Medicine, Department of Radiology, Izmir, Turkey; 2 Dokuz Eylul University Faculty of Medicine, Department of Nuclear Medicine, Izmir, Turkey

**Keywords:** Metastasis, Neuroendocrine tumors, Paget’s disease, PET/CT, Somatostatin, ^68^Ga-DOTANOC

## Abstract

Somatostatin (SST) is a neuropeptide present in neurons, endocrine cells, and a wide range of neuroendocrine tumors (NETs). ^68^Ga-DOTATOC, ^68^Ga-DOTANOC, and ^68^Ga-DOTATATE are current SST analogues used for PET/CT which bind to SST receptors expressed in NETs. These SST analogues have been used successfully for diagnosis of SST-expressing tumors with a more sensitive detection technique than conventional scintigraphy. However, there is a lack of clinical data on the differentiation between NETs and other malignant tumors or benign pathological conditions. Here, we report a case of Paget’s disease mimicking bone metastasis of NET on PET/CT due to increased ^68^Ga-DOTANOC uptake and review examples of similar cases in the literature.

## Introduction

Paget’s disease (osteitis deformans) is a common, chronic, and metabolic skeletal disorder of unknown etiology characterized by disordered and excessive remodeling of bone due to abnormal osseous resorption and nonuniform mineralization [1,2,3]. The disease is often asymptomatic and diagnosed incidentally on radiographs obtained for unrelated causes [4]. When symptomatic, skeletal deformities, pathological fractures resulting in pain, and neuromuscular and cardiovascular complications can be seen [5]. Serum alkaline phosphatase level is used for diagnosis, and its elevation is an indicator of the disease activity [1,3]. Conventional radiography is the major and initial imaging technique for diagnosis of Paget’s disease. Computed tomography (CT) and magnetic resonance imaging may be used when a fracture or sarcomatous degeneration bone is suspected [3]. Bone scintigraphy with ^99m^Tc-labelled bisphosphonate may be more sensitive than conventional radiography in identifying the disease [3]. In up to one-third of patients with Paget’s disease, a variable degree of 2-[^18^F]-fluoro-2-deoxy-D-glucose (FDG) uptake is reported at positron-emission tomography (PET)/CT [6].

Somatostatin (SST) is a neuropeptide present in neurons and endocrine cells which inhibits the secretion of a variety of hormones [7]. It is also present in a wide range of neuroendocrine tumors (NETs) such as carcinoid tumor, pheochromocytoma, renal cell carcinoma, small cell lung cancer, breast cancer, prostate cancer, and malignant lymphoma [8]. ^68^Ga-DOTATOC, Gallium-68 (DOTA0-hel-Tyr3) octreotide (^68^Ga-DOTANOC), and ^68^Ga-DOTATATE are current SST analogues used for PET/CT which bind to SST receptor subtype 2, predominantly expressed in NETs [8]. However, there is a lack of clinical data on the differentiation between NETs and other malignant tumors or benign pathological conditions [9].

In English literature, four cases have been described with increased uptake of different SST analogues due to Paget’s disease. However, to our knowledge, increased ^68^Ga-DOTANOC uptake in a patient with Paget’s disease has not been previously reported. Here, we report a case of Paget’s disease mimicking bone metastasis of NET on PET/CT due to increased ^68^Ga-DOTANOC uptake, which is a somatostatin analogue.

## Case Report

**Figure 1:**
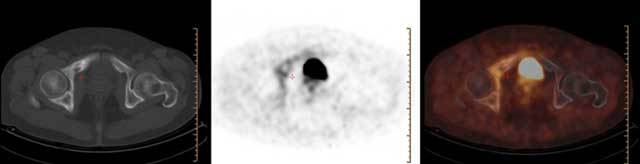
The axial-fused ^68^Ga- DOTANOC PET/CT images show increased uptake in the right pubic bone, superior pubic ramus, and acetabulum.

**Figure 2:**
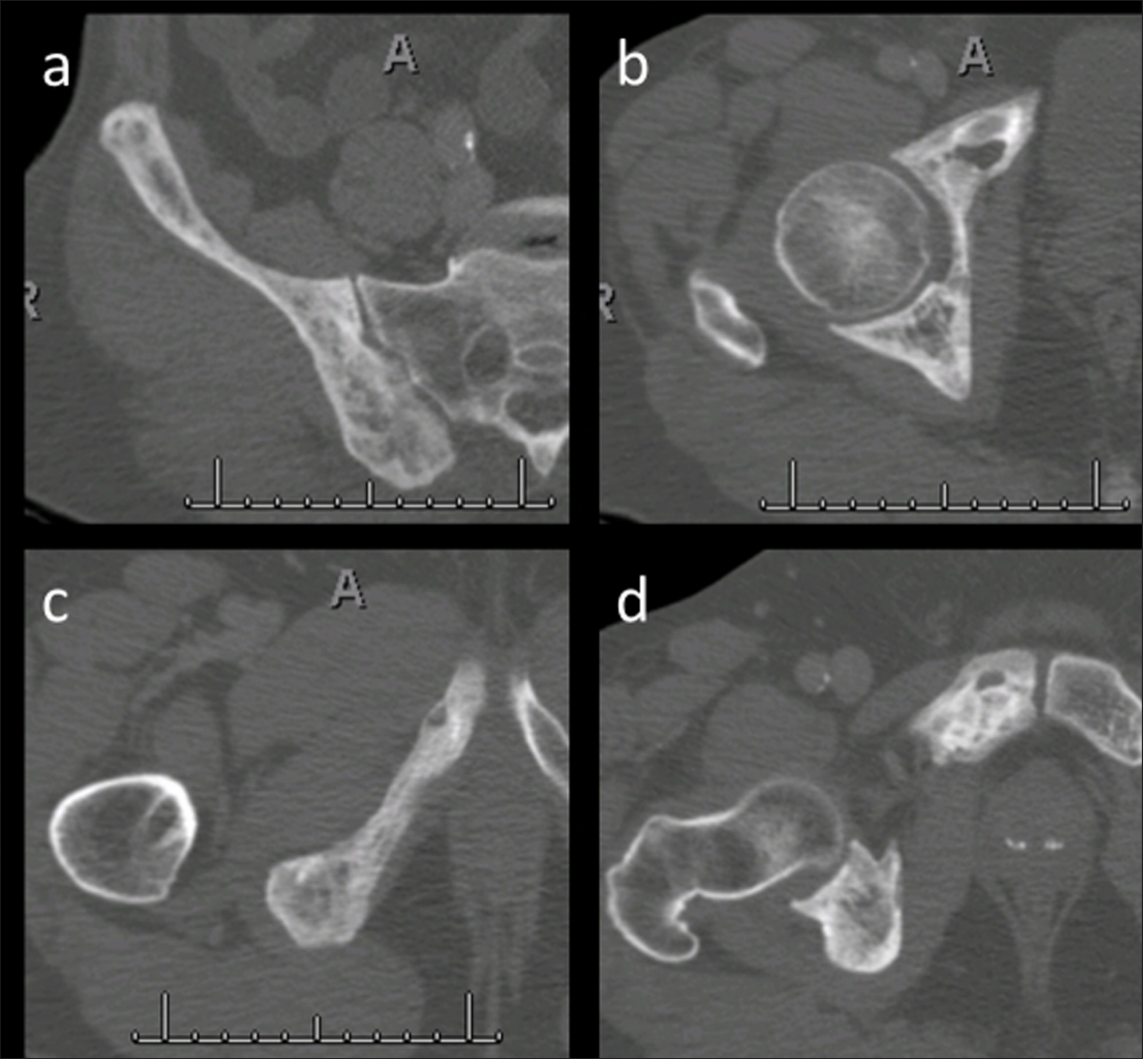
The axial CT images show cortical thickening, trabecular coarsening, and expansion in the right iliac bone (a), acetabulum (b), inferior pubic ramus (c), and pubic bone (d) due to Paget’s disease.

A 65-year old male patient who was diagnosed with gastrointestinal stromal tumor at bulbus of duodenum by gastric endoscopy was referred for a ^68^Ga-DOTANOC PET/CT for investigation of metastasis. PET/CT demonstrated increased tracer uptake in the right ischium, right iliac, and pubic bones (Figure 1). He was asymptomatic without any complaint. Subsequent conventional radiography and CT images confirmed the presence of Paget disease in the right hemi-pelvis (Figure 2).

## Discussion

Since SST has very low metabolic stability, its different gamma- or positron-emitting synthetic analogues have been developed for use in diagnostic applications for SST-expressing tumors [7,10]. These SST analogues have been used successfully for the diagnosis of NETs with a more sensitive detection technique than conventional scintigraphy. However, it is reported that various subtypes of cellular surface somatostatin receptors are described in osteoblasts and other different cell types, such as leukocytes, fibroblasts, and vessels [11].

In differentiation of NETs from other malignant tumors or benign pathological conditions such as Paget’s disease, it should be kept in mind that SST analogues used for PET/CT may lead to false-positive results. In our case, increased ^68^Ga-DOTANOC uptake suspected a metastasis from the NET in the first place. However, radiological and clinical findings led to the diagnosis of Paget’s disease. We suggest that uptake of SST analogues Paget’s disease could be associated with markedly increased bone turnover.

In English literature, four cases with increased uptake of different SST analogues due to Paget’s disease are described. Kang SK et al. [11] reported two cases of In-111 pentetreotide uptake due to Paget’s disease in the humerus and skull. They showed these were the areas of increased osteolytic process. Vandemergel X et al. [12] reported a case of Paget’s disease in the femur associated with phosphate diabetes which had positive octreotide scintigraphy. They hypothesized osteoblasts might manifest somatostatin receptor activity. Minutoli F et al. [13] reported increased ^177^Lu-DOTATATE uptake due to Paget’s disease in the hip bone of a patient who had a surgical resection of a locally invasive NET of ampulla Vater.

## Conclusion

The interpretation of skeletal lesions in a patient with NET might be complicated due to the variable uptake of ^68^Ga-DOTANOC, as in this case. The review of the literature and our case shows Paget’s disease should be kept in mind as a benign differential diagnosis when increased uptake is detected on PET/CT with SST analogues.
